# Asymptomatic sinusitis as an origin of infection-related glomerulonephritis manifesting steroid-resistant nephrotic syndrome: A case report

**DOI:** 10.1097/MD.0000000000020572

**Published:** 2020-06-19

**Authors:** Shohei Noda, Shintaro Mandai, Takashi Oda, Tomoko Shinoto, Hidehiko Sato, Keiko Sato, Katsuiku Hirokawa, Yumi Noda, Shinichi Uchida

**Affiliations:** a Department of Nephrology, Nitobe Memorial Nakano General Hospital, Nakano; b Department of Nephrology, Graduate School of Medical and Dental Sciences, Tokyo Medical and Dental University, Bunkyo; c Department of Nephrology and Blood Purification, Tokyo Medical University Hachioji Medical Center, Hachioji; d Department of Clinical Pathology, Nitobe Memorial Nakano General Hospital, Nakano, Tokyo, Japan.

**Keywords:** asymptomatic infection, glomerulonephritis, nephrotic syndrome, sinusitis

## Abstract

**Rationale:**

Infection is a major trigger or pathogenic origin in a substantial proportion of glomerulonephritis (GN) patients, typically manifesting infection-related GN (IRGN). Various microorganisms, infection sites, and clinical and histopathological features are involved in IRGN. Once an infectious origin is identified and successfully eradicated, nephrotic syndrome or kidney dysfunction is spontaneously resolved. However, if patients are asymptomatic and the origin is undetermined, the diagnosis and treatment of GN is challenging. This case presentation reported on an IRGN case manifesting steroid-resistant nephrotic syndrome associated with asymptomatic sinusitis as a pathogenic origin.

**Patient concerns:**

A 68-year-old male presented with severe kidney dysfunction and edema in both extremities.

**Diagnosis:**

The patient was clinically diagnosed with hypocomplementemic nephrotic syndrome and kidney dysfunction and histopathologically with diffuse proliferative GN and a focal pattern of membranoproliferative GN. The findings suggested that idiopathic membranoproliferative glomerulonephritis type I was more likely than IRGN, given a critical lack of apparent infection.

**Interventions:**

Combined intravenous methylprednisolone, oral prednisolone, and cyclosporin did not improve the patient's condition. Thus, IRGN associated with inapparent infectious origin was suspected. Repeated thorough and careful examinations including CT scan showed sinusitis in his left maxillary sinus. Moreover, reanalysis of kidney specimen revealed positive nephritis-associated plasmin receptor in glomeruli, a typical finding for IRGN, supporting a pathogenic significance of his sinusitis. Medical treatment was initiated with 200 mg oral clarithromycin daily.

**Outcomes:**

Oral clarithromycin gradually improved proteinuria and hypocomplementemia and resulted in nephrotic syndrome remission in parallel with opacification resolution of sinuses shown on CT.

**Lessons:**

This case presentation showed that asymptomatic sinusitis is potentially a pathogenic IRGN origin. A gold standard therapy for idiopathic GN, corticosteroid could be damaging in uncontrolled or underdiagnosed infection. In asymptomatic patients, a thorough screening of infectious diseases, including sinusitis, together with a renal histological evaluation of glomerular nephritis-associated plasmin receptor deposition is also essential in treating a wide spectrum of GN.

## 1 Introduction

Chronic kidney disease (CKD), which eventually progresses to end-stage kidney disease, is a global health problem accounting for 10% to 15% of people.^[1–3]^ CKD is a noncommunicable disease. However, in a substantial number of people with CKD, infectious disease is involved in developing and exacerbating kidney disease with various clinical manifestations (eg, acute kidney injury and electrolyte disorders).^[4,5]^ In particular, much attention has been given to the causal relationship between infection and glomerulonephritis (GN).

Patients with idiopathic GN are partly conventional corticosteroid and/or immunosuppressive treatment resistant; thus, GN is a major reason for end-stage kidney disease.^[6,7]^ Over the past few decades, infectious disease has been recognized as a trigger or pathogenic origin in a number of GN cases, typically infection-related GN (IRGN),^[8–11]^ regardless of microorganisms or infection sites. In IRGN, resolution of infection usually results in partial or complete recovery of kidney function and abnormal urinalysis, whereas immunosuppressive treatment is harmful in uncontrolled infection. Therefore, accurate diagnosis of apparent and inapparent infection is essential in treating all GN patients. However, diagnosis in asymptomatic patients is challenging as well as figuring out if asymptomatic infection is pathogenic in GN.

Here, a case of typical IRGN in an elderly, with a particular exception on asymptomatic sinusitis as a pathogenic source, is reported. Nephrotic syndrome accompanied by hypocomplementemia was resistant to corticosteroid and cyclosporin A (CsA) therapy. However, medical treatment for sinusitis improved GN and eventually led to nephrotic syndrome remission. This case presentation would contribute to deeper understanding on a pathogenic role of asymptomatic or hidden infection in GN and better treatment of a wide spectrum of GN associated with infection. The patient has provided informed consent for publication of the case.

## 2 Case report

**Figure 1:**
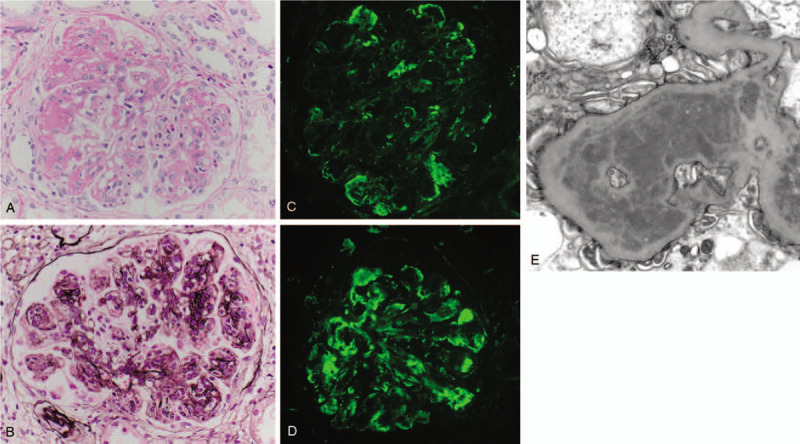
Kidney biopsy findings. (A) Light microscopy (LM) showing diffuse proliferative and exudative glomerulonephritis with infiltrating neutrophils (periodic acid–Schiff; original magnification, × 400). (B) Membranoproliferative pattern showing lobular accentuation and basement membrane duplication was focally evident (periodic acid–methenamine silver; original magnification, × 400). (C, D) Immunofluorescence (IF) microscopy showed granular mesangial and capillary wall deposits for IgG (C) and C3 (D) (original magnification, × 400). (E) Electron microscopy showed paramesangial electron-dense deposits (original magnification, × 10,000).

**Figure 2:**
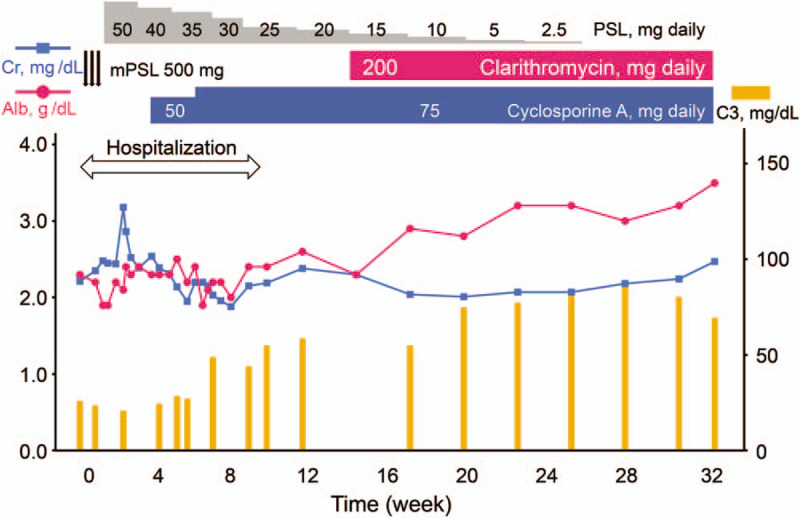
Clinical course of the patient. Alb = serum albumin; C3 = complement C3; Cr = serum creatinine; mPSL= methylprednisolone; PSL = prednisolone. mPSL = methylprednisolone.

**Figure 3:**
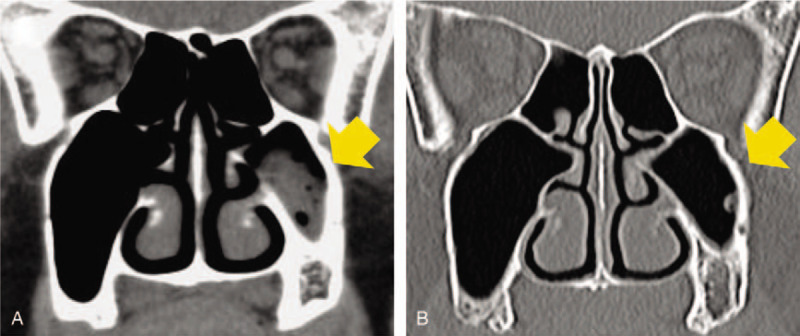
Sinus CT scan of the patient before and after treatment of sinusitis. The mucosal thickening and fluid were found in his left maxillary paranasal sinus (left, arrow), which was substantially improved after medical treatment of sinusitis (right, arrow). CT = computed tomography.

**Figure 4:**
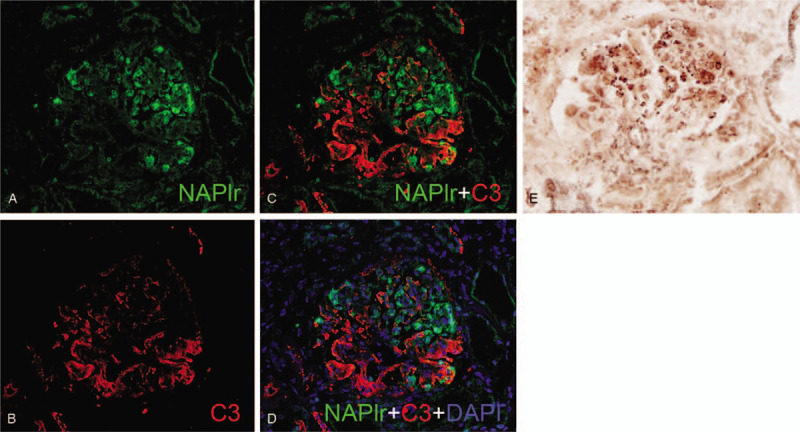
Histological staining for nephritis-associated plasmin receptor (NAPlr) and plasmin activity. (A-D) Double immunofluorescent staining for NAPlr (fluorescein isothiocyanate, green) and complement C3 (Alexa Fluor 594, red) with nuclear staining for DAPI (blue). NAPlr (A) and C3 (B) were both positive in glomeruli, and their localization was essentially different in merged images (C and D). (E) Plasmin activity by in situ zymography was found in a similar distribution as NAPlr staining in glomeruli. DAPI = 4, 6-diamidino-2-phenylindole, NAPlr = nephritis-associated plasmin receptor.

A 68-year-old Japanese man, with about a decade history of non-insulin-dependent type 2 diabetes mellitus, developed edema in lower and upper extremities 1 month prior to hospital admission. He did not report any recent fever, chills, night sweats, skin rashes, and macroscopic hematuria. Medical history also included hypertension and tobacco use. His regular medications were 2 mg trichlormethiazide, 10 mg amlodipine, and 125 mg irbesartan, respectively. On physical examination, the patient was afebrile, pulse rate was 77 beats/min, blood pressure was 154/89 mm Hg, no murmurs or crackles were heard on his chest, and he had bilateral edema on both extremities. Laboratory findings included the following: gross hematuria and proteinuria with protein per gram of creatinine, 7.7; hemoglobin, 10.5 g/dL; white blood cell count, 5,790/mL; platelet count 23.9 × 10^4^/mL; total serum protein, 4.7 g/dL; serum albumin, 2.3 g/dL; urea nitrogen, 39 mg/dL; sCr, 2.21 mg/dL (eGFR, 24 mL/min/1.73 m^2^); sodium, 144 mEq/L; potassium, 4.4 mEq/L; chloride, 114 mEq/L; hemoglobin A1c, 6.5%; and C3-dominant hypocomplementemia (Table 1). Pertinent antibodies associated with GN were negative (Table 1). Serum and urine electrophoresis and serum free light chain levels were unremarkable. Chest radiography showed no pulmonary congestion or pleural effusions and echocardiography no regurgitation or vegetations. A kidney biopsy specimen showed 32 glomeruli, nine of which were globally sclerotic. Glomeruli showed diffuse proliferative and exudative GN with neutrophil infiltrates (Fig. 1A). Focal lobular accentuation and double contour of the capillary wall, typical of membranoproliferative GN (MPGN) type I (Fig. 1B), moderate to severe interstitial fibrosis, and arterial wall thickening in the inner layer were observed. Immunofluorescence (IF) microscopy showed granular mesangial and capillary wall deposits for IgG (1+) and C3 (2+) (Fig. 1C and D). IgA, IgM, C4, or C1q staining was not noted. Paramesangial dense deposits (Fig. 1E) were found on electron microscopy, but subepithelial deposits or humps were not evident. The histological findings suggested idiopathic MPGN because of a lack of apparent infection and typical humps on electron microscopy. Daily 500 mg intravenous methylprednisolone for 3 days followed by 50 mg oral prednisolone was started (Fig. 2). Given the poor response to steroids, oral CsA 75 mg per day was added on day 57, and the patient was discharged on day 67. The effect of the addition of CsA was limited, resulting in a slight increase in complement C3 and no improvement in proteinuria and serum albumin (Fig. 2). Thus, IRGN caused by a hidden infectious origin was reconsidered. After the repeated examinations and diagnostic tests including CT scan, mucosal thickening and fluid in the left maxillary paranasal sinus were detected (Fig. 3). The patient's sinusitis remained asymptomatic even when diagnosed. Glomerular staining of nephritis-associated plasmin receptor (NAPlr) was also evaluated, typically positive in IRGN.^[11,12]^ Deposition of NAPlr and related plasmin activity were observed in similar distributions in glomeruli (Fig. 4), suggesting that asymptomatic sinusitis contributed to the onset of GN. Thus, low-dose continuous administration of macrolide (clarithromycin 200 mg once daily) was started instead of a surgical treatment, after the patient's comprehension of the disease and treatment options. As a result of this treatment under good adherence and tolerance, proteinuria gradually decreased and serum albumin increased up to 3.5 g/dL, resulting in nephrotic syndrome remission 2 months after clarithromycin administration (Fig. 2). In parallel, complement gradually increased to the normal range. His sinusitis was substantially improved as shown in the repeated CT scan (Fig. 3).

**Table 1:**
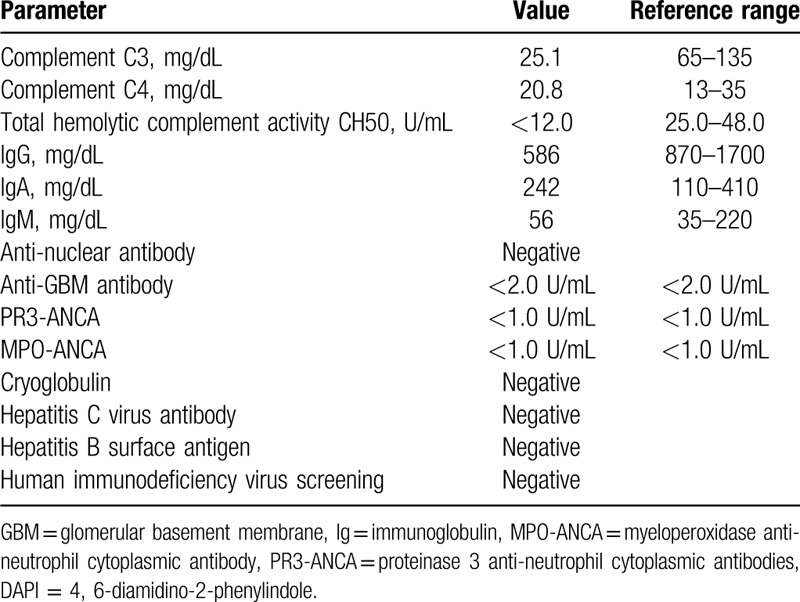
Serologic tests associated with glomerulonephritis on admission.

## 3 Discussion

In this case presentation, an elderly man developed GN presenting with nephrotic syndrome and C3-dominant hypocomplementemia that were corticosteroid and immunosuppressive treatment resistant. Oral clarithromycin for asymptomatic sinusitis gradually improved proteinemia in parallel with the increases in serum albumin and complement levels, resulting in nephrotic syndrome remission. Thorough analysis of this case revealed the pathogenic relationship between GN and sinusitis, even if asymptomatic. This report is the first to reveal asymptomatic sinusitis as a pathogenic origin of IRGN.

IRGN was earlier known to follow a streptococcal infection at the upper respiratory tract or soft tissues and was called postinfectious GN. Over the previous decades, this disease entity has been shifted particularly in terms of diversity of the causative mircroorganism and origin.^[8–11]^ In the present case, GN was associated with sinusitis and the typical histological findings for IRGN, showing diffuse proliferative GN with predominant C3 and IgG depositions on IF. The MPGN pattern focally seen in the patient's histopathology was consistent with IRGN, given that 2% to 8% of IRGN patients develop MPGN.^[10,11,14]^ A case of MPGN following bilateral sinusitis is also reported,^[15]^ and immunosuppressive treatment provided little effect, leading to an unfavorable kidney outcome. Thus, in our case, added workups were repeated and CT scan showed asymptomatic sinusitis. The previous case series of IRGN or postinfectious GN included patients with unknown source of infection with and without septicemia.^[11,16]^ Thus, there would be much more GN cases with a hidden infectious origin than currently expected.

Positive glomerular NAPlr staining and plasmin activity in this case support IRGN diagnosis. This finding led to the treatment of his sinusitis with medications preceding surgical treatment. This resulted in GN remission and determined the pathogenetic significance of his sinusitis. NAPlr is a nephritogenic protein that was originally isolated from group A streptococcus and shows high homology in nucleotide and amino acid sequences to streptococcal plasmin receptor.^[12,13]^ NAPlr induces glomerular damages by trapping plasmin and maintaining its activity, and glomerular NAPlr deposition and related plasmin activity are usually detected in classical postinfectious GN. However, these findings are increasingly reported irrespective of microorganisms or morphological types.^[17–20]^ In such cases, infection resolution resulted in complete or incomplete remission of GN, similar to our case. IF staining for NAPlr and in situ zymography for plasmin activity on kidney biopsy tissues could be a key diagnostic tool in possible IRGN with unknown source of infection.

This case showed that asymptomatic sinusitis could be a pathophysiological IRGN cause. Several reports including non-English literatures have documented that symptomatic sinusitis treatment resulted in nephrotic syndrome remission in various types of GN such as membranous nephropathy^[21]^ and minimal change disease.^[22]^ Of interest is that the patient remained asymptomatic from the initial presentation until the sinusitis remission. Sinusitis is an inflammatory disease of the paranasal sinuses, annually affecting approximately 16% of the adult population.^[23]^ This condition typically causes cardinal symptoms including facial pain/pressure or nasal obstruction and decrease quality of life. The population is increasing, and acute/chronic/recurrent sinusitis is currently a healthcare burden.^[23,24]^ Previous epidemiologic studies included only symptomatic patients, and routine medical check-ups could not easily identy sinusitis especially when asymptomatic. Thus, the true incidence of sinusitis and sinusitis-associated GN may be much higher than reported.

The classical disease concept of postinfectious GN or IRGN has been increasingly expanded.^[8–10,16,25]^ A relative disease of IRGN is IgA-dominant IRGN, alternatively called staphylococcus-associated GN, showing diffuse endocapillary proliferative and exudative GN with predominant IgA deposition.^[26,27]^ We previously reported and overviewed post-staphylococcal infection Henoch-Schönlein purpura nephritis (HSPN), alternatively called staphylococcus-associated HSPN.^[28]^ This GN's clinical and morphological characteristics are closely similar with those of IgA-dominant IRGN, rather than those of HSPN.^[28]^ Moreover, improvement of proteinuria or kidney function after controlling infection is not limited to GN but also to various glomerular diseases (eg, minimal change disease, membranous nephropathy, and proliferative GN with monoclonal IgG deposits),^[18,21,22]^ indicating the pathogenic role of infection in a wider spectrum of glomerular diseases. “Infection-related glomerulopathy” may be adopted to characterize a broader domain of such diseases. This case presentation aimed to contribute in understanding the concept of a pathogenic role of infection and the need for screening in extensive glomerular diseases.

Therefore, an IRGN case associated with asymptomatic sinusitis manifesting nephrotic syndrome and hypocomplementemia resistant to steroid with immunosuppressant was documented. Medical treatment of sinusitis resulted in nephrotic syndrome remission.

## Acknowledgments

We would like to thank Prof. Yoshihiko Ueda (Department of Pathology, Dokkyo Medical University Koshigaya Hospital) for kind and helpful discussions about pathological findings of the patient.

## Author contributions

S.N., S.M., T.S., H.S., K.S. participated in patient care, acquisition of data, and writing the manuscript. S.M. supervised and substantively revised the manuscript. T.O. contributed to IF staining for NAPlr and and in situ zymography for plasmin activity on kidney biopsy tissues. T.O., K.H., Y.N., and S.U. participated in discussions and critical review of the manuscript.

## References

[1] Coresh J Selvin E Stevens LA Prevalence of chronic kidney disease in the United States. JAMA 2007;298:2038–47. 17986697 10.1001/jama.298.17.2038

[2] Murphy D McCulloch CE Lin F Centers for Disease Control and Prevention Chronic Kidney Disease Surveillance Team. Trends in prevalence of chronic kidney disease in the United States. Ann Intern Med 2016;165:473–81. 27479614 10.7326/M16-0273 PMC5552458

[3] Imai E Horio M Watanabe T Prevalence of chronic kidney disease in the Japanese general population. Clin Exp Nephrol 2009;13:621–30. 19513802 10.1007/s10157-009-0199-x

[4] Couser WG Johnson RJ The etiology of glomerulonephritis: roles of infection and autoimmunity. Kidney Int 2014;86:905–14. 24621918 10.1038/ki.2014.49

[5] Prasad N Patel MR Infection-induced kidney diseases. Front Med (Lausanne) 2018;5:327. 30555828 10.3389/fmed.2018.00327 PMC6282040

[6] Imai E Yamagata K Iseki K Kidney disease screening program in Japan: history, outcome, and perspectives. Clin J Am Soc Nephrol 2007;2:1360–6. 17942780 10.2215/CJN.00980207

[7] United States Renal Data System. 2015 USRDS annual data report: Epidemiology of Kidney Disease in the United States. National Institutes of Health, National Institute of Diabetes and Digestive and Kidney Diseases, Bethesda, MD, 2015. Available online at https://www.usrds.org/2015/view/.

[8] Nasr SH Radhakrishnan J D’Agati V D Bacterial infection-related glomerulonephritis in adults. Kidney Int 2013;83:792–803. 23302723 10.1038/ki.2012.407

[9] Satoskar AA Parikh SV Nadasdy T Epidemiology, pathogenesis, treatment and outcomes of infection-associated glomerulonephritis. Nat Rev Nephrol 2020;16:32–50. 31399725 10.1038/s41581-019-0178-8

[10] Montseny JJ Meyrier A Kleinknecht D Callard P The current spectrum of infectious glomerulonephritis. Experience with 76 patients and review of the literature. Medicine (Baltimore) 1995;74:63–73. 7891544 10.1097/00005792-199503000-00001

[11] Nasr SH Markowitz GS Stokes MB Said SM Valeri AM D’Agati VD Acute postinfectious glomerulonephritis in the modern era: experience with 86 adults and review of the literature. Medicine (Baltimore) 2008;87:21–32. 18204367 10.1097/md.0b013e318161b0fc

[12] Oda T Yamakami K Omasu F Glomerular plasmin-like activity in relation to nephritis-associated plasmin receptor in acute poststreptococcal glomerulonephritis. J Am Soc Nephrol 2005;16:247–54. 15574512 10.1681/ASN.2004040341

[13] Oda T Yoshizawa N Yamakami K Localization of nephritis-associated plasmin receptor in acute poststreptococcal glomerulonephritis. Hum Pathol 2010;41:1276–85. 20708459 10.1016/j.humpath.2010.02.006

[14] Moroni G Pozzi C Quaglini S Long-term prognosis of diffuse proliferative glomerulonephritis associated with infection in adults. Nephrol Dial Transplant 2002;17:1204–11. 12105242 10.1093/ndt/17.7.1204

[15] Lorcy N Rioux-Leclercq N Lombard ML Le Pogamp P Vigneau C Three kidneys, two diseases, one antibody? Nephrol Dial Transplant 2011;26:3811–3. 21813829 10.1093/ndt/gfr436

[16] Nasr SH Fidler ME Valeri AM Postinfectious glomerulonephritis in the elderly. J Am Soc Nephrol 2011;22:187–95. 21051737 10.1681/ASN.2010060611

[17] Komaru Y Ishioka K Oda T Ohtake T Kobayashi S Nephritis-associated plasmin receptor (NAPlr) positive glomerulonephritis caused by aggregatibacter actinomycetemcomitans bacteremia: a case report. Clin Nephrol 2018;90:155–60. 29578396 10.5414/CN109173

[18] Takehara E Mandai S Shikuma S Post-infectious proliferative glomerulonephritis with monoclonal immunoglobulin G deposits associated with complement factor H mutation. Intern Med 2017;56:811–7. 28381748 10.2169/internalmedicine.56.7778 PMC5457925

[19] Odaka J Kanai T Ito T A case of post-pneumococcal acute glomerulonephritis with glomerular depositions of nephritis-associated plasmin receptor. CEN Case Rep 2015;4:112–6. 28509278 10.1007/s13730-014-0149-7 PMC5413717

[20] Yamashiro A Uchida T Ito S Oshima N Oda T Kumagai H Complete remission of minimal change disease following an improvement of lung Mycobacterium avium infection. Intern Med 2016;55:2669–72. 27629965 10.2169/internalmedicine.55.6885

[21] Kanazawa I Yano S Takase H Yamane Y Yamaguchi T Sugimoto T A case of membranous nephropathy associated with chronic sinusitis. J Nephrol 2009;22:289–94. 19384848

[22] Iwatani H Mori D Yamamoto S Minimal change nephrotic syndrome which was most likely caused by chronic sinusitis. Intern Med 2015;54:2373–5. 26370864 10.2169/internalmedicine.54.4480

[23] Anand VK Epidemiology and economic impact of rhinosinusitis. Ann Otol Rhinol Laryngol Suppl 2004;193:3–5. 15174752 10.1177/00034894041130s502

[24] Chung SD Hung SH Lin HC Lin CC Health care service utilization among patients with chronic rhinosinusitis: a population-based study. Laryngoscope 2014;124:1285–9. 24338913 10.1002/lary.24500

[25] Glassock RJ Alvarado A Prosek J Staphylococcus-related glomerulonephritis and poststreptococcal glomerulonephritis: why defining “post” is important in understanding and treating infection-related glomerulonephritis. Am J Kidney Dis 2015;65:826–32. 25890425 10.1053/j.ajkd.2015.01.023

[26] Haas M Racusen LC Bagnasco SM IgA-dominant postinfectious glomerulonephritis: a report of 13 cases with common ultrastructural features. Hum Pathol 2008;39:1309–16. 18619648 10.1016/j.humpath.2008.02.015

[27] Nasr SH D’Agati VD IgA-dominant postinfectious glomerulonephritis: a new twist on an old disease. Nephron Clin Pract 2011;119:c18–25. 21659781 10.1159/000324180

[28] Mandai S Aoyagi M Nagahama K Post-staphylococcal infection Henoch-Schönlein purpura nephritis: a case report and review of the literature. Ren Fail 2013;35:869–74. 23721509 10.3109/0886022X.2013.794703

